# Precision targeting of STING: Challenges, innovations, and clinical outlook for cancer therapy

**DOI:** 10.1016/j.xinn.2025.101074

**Published:** 2025-08-06

**Authors:** Jiaqi Shi, Yingying Zhang, Na Zhao, Ekihiro Seki, Li Ma, Gordana Kocic, Xiaobo Li, Janoš Terzić, Tongsen Zheng

**Affiliations:** 1 Department of Phase I Trials Center, Harbin Medical University Cancer Hospital, 150 Haping Road, Nangang District, Harbin 150081, P.R. China; 2 Heilongjiang Province Key Laboratory of Molecular Oncology, 150 Haping Road, Nangang District, Harbin 150081, P.R. China; 3 Department of Gastrointestinal Medical Oncology, Harbin Medical University Cancer Hospital, 150 Haping Road, Nangang District, Harbin 150081, P.R. China; 4 Karsh Division of Gastroenterology and Hepatology, Cedars-Sinai Medical Center, Los Angeles, CA 90048, USA; 5 Samuel Oschin Comprehensive Cancer Institute, Cedars-Sinai Medical Center, Los Angeles, CA 90048, USA; 6 Department of Experimental Radiation Oncology, The University of Texas MD Anderson Cancer Center, Houston, TX 77030, USA; 7 Department of Biochemistry, Faculty of Medicine, University of Nis, 18000 Nis, Serbia; 8 Department of Pathology, Harbin Medical University, 157 Baojian Road, Nangang District, Harbin 150081, P.R. China; 9 Laboratory for Cancer Research, University of Split School of Medicine, Split, 21000 Split, Croatia

**Keywords:** STING, cancer, immunotherapy, clinical translation, resistance

## Abstract

The stimulator of interferon genes (STING) pathway plays a crucial role in immune responses and has emerged as a compelling target in cancer therapy. Despite promising preclinical studies, clinical trials of STING agonists have largely failed to deliver durable efficacy, with no agents progressing to phase III trials. This review examines the biological, pharmacological, and clinical barriers limiting STING pathway activation in cancer treatment. We discuss the inherent limitations of STING agonists as well as host-related resistance driven by tumor heterogeneity, immune suppression, and chronic STING activation. Mechanisms of acquired resistance, such as immune checkpoint upregulation and suppression of effector immune cells, are also reviewed. Recent advances in delivery platforms, small-molecule design, and combination treatment strategies offer promising paths forward. We highlight precision approaches based on human STING variants, epigenetic modulation, and biomarker-driven patient stratification to improve clinical outcomes. These insights underscore the need for refined, context-specific STING activation strategies to unlock the full therapeutic potential of this pathway in oncology.

## Public summary

• The STING agonists have yet to demonstrate durable clinical efficacy.
• Drug limitations, tumor heterogeneity, and immune suppression contribute to treatment resistance.
• Drug delivery, molecular design, and combination strategies offer promising clinical translation avenues.
• This review outlines the barriers to STING activation and the strategies for STING-targeted immunotherapy.

## Graphical abstract

**Figure:**
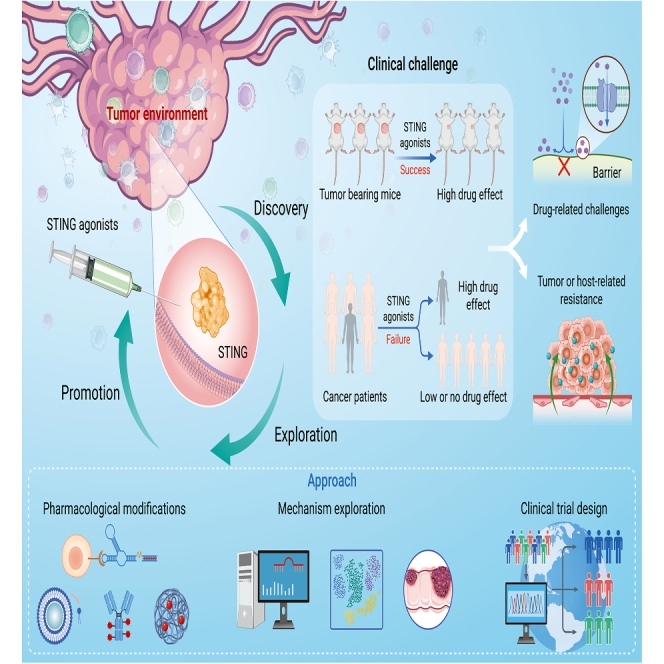

## Introduction

The stimulator of interferon genes (STING) is a key protein in the innate immune response to cytoplasmic pathogenic DNA,^1^^,^^2^ activated by cyclic GMP-AMP (cGAMP) synthesized by cyclic GMP-AMP synthase (cGAS).^3^^,^^4^^,^^5^^,^^6^ Upon activation, STING phosphorylates TANK-binding kinase 1 (TBK1), which subsequently activates interferon regulatory factor 3 (IRF3), leading to the expression of type I interferons (IFNs) and chemokines.^3^^,^^7^^,^^8^^,^^9^^,^^10^^,^^11^^,^^12^ This signaling cascade promotes dendritic cell (DC) activation and enhances antigen presentation, further stimulating CD8^+^ T cell recruitment and activation to potentiate anti-tumor immunity.^3^^,^^13^^,^^14^ Due to these effects, STING is increasingly recognized as a promising target in cancer immunotherapy.^4^^,^^15^ STING agonists have been extensively developed,^16^^,^^17^^,^^18^^,^^19^ and have shown substantial tumor-suppressive effects in multiple preclinical models of malignancy, with some even achieving tumor regression.^20^^,^^21^^,^^22^^,^^23^

**Figure 1:**
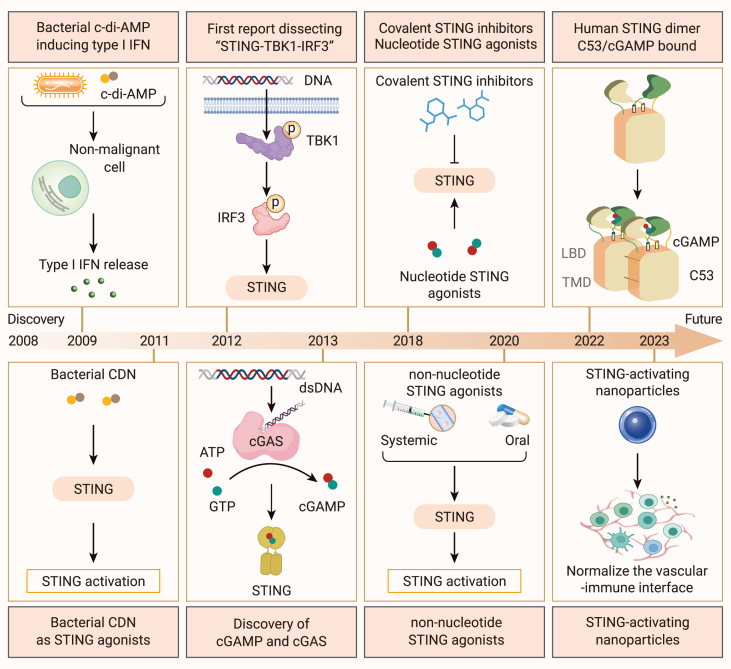
A timeline of STING development Timeline depicting the brief history of STING discovery and key events about STING biology and agonist applications since its identification in 2008.

In recent years, the exploration of STING has progressed significantly, yielding a wealth of groundbreaking findings. These discoveries have provided strong impetus for the development of novel STING agonists. In 2019, researchers led by Shang first disclosed the cryoelectron microscopy structure of full-length STING.^24^ Three years later, they revealed the existence of a second agonist-binding site in the transmembrane region of STING,^25^ providing a structural basis for the development of new STING-targeted drugs. Wang-Bishop et al., using the STING-activating nanoparticles they developed, have demonstrated that STING agonists can promote the normalization of tumor vasculature, ameliorate the hypoxic microenvironment, enhance the expression of endothelial cell adhesion molecules, and recruit more immune effector cells, thereby exerting an inhibitory effect on tumor growth (Figure 1).^26^

Despite the promising results observed in preclinical studies, the clinical efficacy of STING agonists remains limited, with unclear underlying mechanisms representing a major barrier to success.^27^^,^^28^^,^^29^ Additionally, resistance to STING agonists has emerged as an important concern. Investigating resistance mechanisms and identifying sensitization strategies are vital for addressing translational challenges. Here, we review the factors underlying the limited clinical efficacy of STING agonists, examining both intrinsic properties of STING agonists and tumor-specific characteristics (primary and acquired resistance). We further discuss current solutions and future directions aiming at overcoming these therapeutic barriers.

## Barriers to STING agonist efficacy

### Drug-related challenges

#### Limited membrane permeability

**Figure 2:**
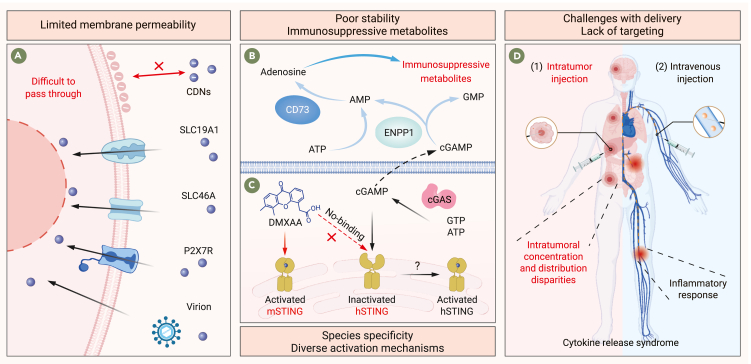
Factors limiting the efficacy of STING agonists (A) CDNs struggle to bind with STING due to membrane permeability barriers, and specific transporters and viral particles among other mechanisms can help overcome these barriers, but there are still many limitations. (B) CDNs exhibit poor stability and are readily hydrolyzed by ENPP1 into immunosuppressive metabolites. (C) DMXAA exhibits species specificity and cannot bind to hSTING. The conformational changes and activation mechanisms of STING still need to be explored. (D) Both intratumoral injection and intravenous administration lack tumor and cellular specificity targeting. Intratumoral administration faces the issues of concentration differences within the tumor, and the heterogeneity in tumor location, size, and morphology poses significant challenges. Intravenous administration can easily trigger inflammatory responses and other side effects.

STING is a transmembrane protein primarily localized in the endoplasmic reticulum (ER).^30^ Due to their negative charge, strong polarity, and high water solubility, natural STING agonist cyclic dinucleotides (CDNs) struggle to penetrate cell membranes and bind to STING, thus limiting their bioactivity.^31^^,^^32^^,^^33^^,^^34^ Previous studies have identified specific transporters, such as SLC19A1, SLC46A, and P2X7R, that facilitate the intracellular entry of cGAMP.^35^^,^^36^^,^^37^ Additional mechanisms, including gap junctions, volume-regulated anion channel (VRAC) channels, viral particles, and cellular endocytosis, have also assisted cGAMP in crossing the lipid bilayer.^38^^,^^39^^,^^40^ Zhang and co-workers further demonstrated that the human host defense peptide LL-37 can act as a cGAMP transporter, enhancing STING-mediated innate immunity.^41^ Nonetheless, the efficiency of cGAMP uptake remains low,^21^^,^^38^ and the expression of cGAMP transporters may be influenced by cell type, cellular state, and environmental factors.^42^^,^^43^^,^^44^ Therefore, overcoming the membrane permeability barrier is essential to fully realize the biological potential of STING agonists (Figure 2A).

#### Poor stability and immunosuppressive metabolites

In addition to limited cell permeability, CDNs suffer from poor metabolic stability and extremely short half-lives after intravenous administration.^21^^,^^31^ Ectonucleotide pyrophosphatase/phosphodiesterase I (ENPP1) can hydrolyze cGAMP directly, producing AMP and GMP, and can also hydrolyze ATP to AMP.^32^^,^^45^ AMP derived from both pathways is rapidly dephosphorylated by CD73 into adenosine, which suppresses immune cell function and promotes tumor metastasis.^46^^,^^47^ This suggests that ENPP1 can shift the immunostimulatory effect of STING agonists toward immunosuppression (Figure 2B).

#### Species specificity of STING agonists

5,6-Dimethylxanthenone-4-acetic acid (DMXAA, ASA404), one of the first non-CDN small-molecule STING agonists, exhibited anti-tumor and immunomodulatory effects in murine models.^48^ However, subsequent clinical studies revealed that DMXAA selectively binds to mouse STING (mSTING) and fails to bind human STING (hSTING).^49^^,^^50^ This finding underscores the importance of carefully considering species specificity in the development of STING agonists and rigorously validating their ability to bind and activate hSTING through clinical trials (Figure 2C).^7^^,^^34^

#### Diverse activation mechanisms of STING agonists

It is widely accepted that STING transitions from an “open” to a “closed” conformation upon activation, a structural change associated with CDN binding.^51^ However, diABZI, another STING agonist, activates STING while maintaining it in the open conformation.^23^ This unique conformation may represent a novel mechanism of STING activation. Further research into the structural dynamics and activation mechanisms of STING could inspire new directions for the development of STING agonists (Figure 2C).^52^

#### Challenges in intratumoral delivery

While systemic administration of STING agonists poses risks of cytokine storms, intratumoral injection offers a targeted approach with reduced systemic exposure.^53^ Despite its promising potential in solid tumor treatment, intratumoral CDN delivery faces several challenges: first, tumor heterogeneity across different cancer types and individual patients makes it difficult to standardize administration protocols. Additionally, variations in tumor location, size, morphology, vascularization, and mechanical properties, along with the rapid diffusion of CDNs post-injection, lead to significant disparities in intratumoral concentration and distribution, which can trigger heterogeneous responses across different tumor regions and complicate dose optimization.^54^ Furthermore, intratumoral injection requires advanced imaging techniques for precise guidance, which may not always be feasible; and due to the short half-life of STING agonists such as dithio-(RP,RP)-[cyclic[A(2′,5′)pA(3′,5′)p]] (ADU-S100) (10–20 min), multiple injections are often required to achieve therapeutic efficacy, posing additional challenges in clinical practice.^55^ Lastly, intratumoral delivery cannot address issues related to common tumor recurrence and metastasis. Therefore, significant technical and biological hurdles must be overcome to enable the clinical application of intratumoral STING agonists (Figure 2D).

#### Lack of tumor and cellular targeting

Optimal activation of STING should ideally occur within the tumor, creating a local inflammatory environment that releases chemokines to recruit T cells into the tumor microenvironment (TME), while limiting activation in the bloodstream and other tissues. A major challenge of systemic STING agonist administration is the lack of tumor and cell-specific targeting.^55^ Systemic delivery inevitably results in off-target effects in normal tissues, leading to systemic inflammatory responses, potentially causing severe cytokine release syndrome (CRS) with symptoms such as fatigue, fever, headache, and multi-organ failure.^55^ Additionally, systemic activation could trigger autoimmune diseases, tissue toxicity, or even create a pro-tumor inflammatory microenvironment.^3^^,^^56^ Thus, developing STING agonists with targeted delivery to tumors is urgently needed to enhance immune activation while minimizing side effects (Figure 2D).

### Tumor- or host-related resistance

#### Differential responsiveness of hSTING variants to STING agonists

**Figure 3:**
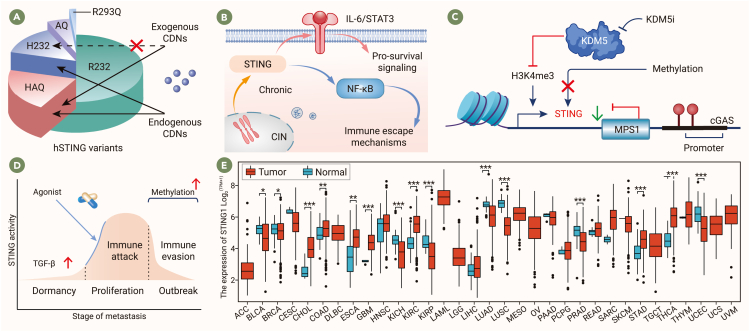
Host factors driving primary resistance to STING agonists (A) hSTING exists in five major variants with varying responsiveness to STING agonists. (B) CIN in tumors leads to chronic STING activation, which may contribute to immune evasion and worse clinical outcomes. (C) Tumor cells often suppress the cGAS-STING pathway to evade immune surveillance with epigenetic mechanisms. (D) STING expression fluctuates during tumor metastasis and STING agonists may enhance immune responses during early metastasis. (E) Expression profile of the TMEM173 gene in tumor samples and paired normal tissues from the TCGA database. Data are represented as mean ± SEM. A one-way ANOVA with Tukey’s multiple comparisons test (E) was used. *∗p <* 0.05, *∗∗p <* 0.01, *∗∗∗p <* 0.001.

hSTING displays single-nucleotide polymorphisms and exists in five major variants: R232 (57.9%), HAQ (20.4%), H232 (13.7%), AQ (5.2%), and R293Q (1.5%).^57^ Sensitivity to STING agonists varies among these variants. For instance, STING-H232 responds to endogenous 2′,3′-cGAMP but not to certain exogenous CDNs, whereas STING-HAQ shows reduced responsiveness to both exogenous and endogenous CDNs (Figure 3A).^57^^,^^58^ Additionally, the distribution of these variants differs by region,^57^^,^^59^ with the HAQ variant being common in East Asian populations but rare in African populations.^60^ This underscores the importance of considering hSTING variants and their geographic distribution when enrolling participants in clinical trials to enhance the scientific rigor and generalizability of results.

#### Chronic STING activation in CIN-high tumors diminishes response to STING agonists

The role of tumor-intrinsic STING activation in cancer progression remains controversial. While STING activation is generally associated with anti-tumor immunity and apoptosis, high STING expression in tumors often predicts better prognosis across cancers.^61^^,^^62^ However, in breast and ovarian cancer patients undergoing adjuvant chemotherapy, high STING expression, particularly in proliferative cells, is linked to an increased recurrence risk.^63^ In advanced and metastatic tumors, chromosomal instability (CIN) escalates with tumor progression,^64^ leading to chronic STING activation,^65^ which drives IL-6-STAT3 signaling and fosters CIN-positive tumor cell survival.^66^ High CIN tumor models in mice demonstrate more severe metastasis and lower survival rates due to NF-κB activation through the non-canonical STING pathway, promoting immune escape and metastasis.^67^^,^^68^^,^^69^ Studies show that repeated STING agonist stimulation in cells initially triggers a strong IFN response but, over time, it would suppress IFN signaling and activate ER stress and NF-κB-related genes, aiding immune evasion.^65^ Chronic STING activation may thus contribute to poor outcomes with the application of STING agonists in patients with advanced or metastatic tumors (Figure 3B).

#### Suppression of the cGAS-STING pathway in tumor cells

Tumor cells often display alterations in DNA damage response (DDR) systems and amplified CIN.^70^ DDR is a highly conserved mechanism that protects the genome from damage. In tumor cells, mutations in genes encoding DDR components result in defective DNA repair and CIN,^70^ which involves errors in chromosome segregation that produce aneuploid cells and micronuclei. In tumors with high CIN, micronuclei may rupture, releasing DNA and activating the cGAS-STING pathway.^71^ To evade STING-driven immune surveillance, tumor cells often adopt epigenetic mechanisms to block STING activation as a survival strategy.^72^^,^^73^^,^^74^

For example, STING expression is suppressed in pancreatic ductal adenocarcinoma (PDAC) cells, contributing to an immune-cold TME.^75^ In glioblastoma, STING expression is downregulated in tumor cells, although it remains stable in tumor-associated immune and stromal cells.^76^^,^^77^^,^^78^ Similarly, hepatocellular carcinoma (HCC) cells exhibit reduced STING expression compared with non-tumor tissue, which may correlate with impaired immune surveillance.^21^ Melanoma cells epigenetically silence cGAS or STING, thereby avoiding type I IFN production and immune cytokine release after cytoplasmic DNA exposure.^79^^,^^80^

Mechanistically, STING signaling in tumor cells is frequently inhibited due to promoter mutations or hypermethylation of cGAS and STING.^72^^,^^73^^,^^79^^,^^81^^,^^82^^,^^83^ STING expression is also regulated by histone modifications; the histone H3 Lys4 demethylases KDM6B and KDM5C suppress STING expression.^84^ Additionally, the endogenous promoter region of the TMEM173 gene has binding sites for the transcription factor MYC, and MYC knockout in triple-negative breast cancer leads to TMEM173 upregulation, increased CXCL10 and CCL5 production, and enhanced CD8^+^ T cell infiltration.^85^^,^^86^ Tumor cells may also inhibit STING activation by upregulating certain genes that stabilize the genome and prevent micronucleus formation, such as monopolar spindle 1 (MPS1), which plays a critical role in mitosis and is associated with various cancers.^87^ Inhibiting MPS1 induces micronucleus formation and directly activates STING.^88^ Furthermore, tumors with IDH1 mutation show selective cGAS hypermethylation and silencing; mIDH1 inhibition reactivates cGAS expression and certain transposable elements (Figure 3C).^89^

#### Changes in STING expression during different stages of tumor metastasis

STING expression dynamically fluctuates during tumor metastasis.^90^ In the early dormancy stage of metastasis, low STING activity enables cancer cells to evade immune surveillance. As dormant cells exit dormancy and initiate proliferation, STING activity increases, making these cells more susceptible to immune attack. Surviving cancer cells form larger metastases, again exhibiting low STING levels and enhanced immune evasion. Mechanistically, high TGF-β expression suppresses STING expression during dormancy, while promoter and enhancer methylation downregulates STING expression in metastatic outbreaks.^90^

Thus, STING upregulation in proliferative cancer cells during early metastasis may allow immune recognition and containment of these cells, limiting progression from dormancy to recurrence (Figure 3D).^90^ Preclinical studies indicate that STING agonists used in early metastasis stages promote natural killer (NK) cell and T cell recruitment, enhancing cytotoxicity against metastatic cancer cells and suppressing metastatic outgrowth.^90^

#### Variations in STING expression across cancer types

STING expression varies significantly across cancers. For instance, TMEM173, the gene encoding STING, is upregulated in colorectal cancer, kidney clear cell carcinoma, thyroid cancer, and gastric adenocarcinoma, while it is downregulated in uterine corpus endometrial carcinoma, prostate cancer, lung squamous cell carcinoma, and lung adenocarcinoma.^91^ These differences could imply varying sensitivities to STING agonists across cancer types and subtypes (Figure 3E). Additionally, intratumoral heterogeneity in cell composition and STING expression complicates efficacy assessments for STING agonists.^54^

#### Overexpression of secondary immune checkpoint molecules

**Figure 4:**
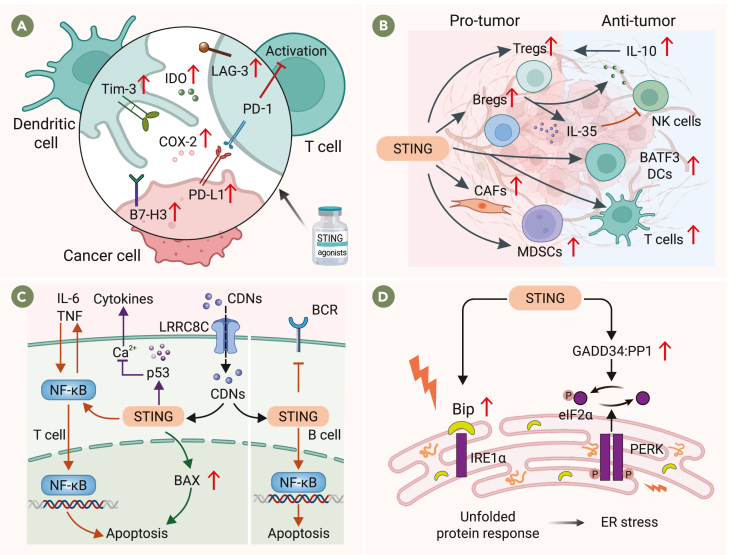
Mechanisms of acquired resistance mediated by cGAS-STING pathway activation (A) cGAS-STING pathway activation can lead to the upregulation of immune checkpoint molecules. (B) STING activation can enhance the presence of immunosuppressive cells in the TME. (C) Excessive STING activation may lead to suppression of T and B cell function by reducing their proliferation, infiltration, and promoting apoptosis. (D) ER stress leads to the accumulation of misfolded proteins.

The sustained activation of the cGAS-STING pathway is associated with the upregulation of indoleamine 2,3-dioxygenase (IDO), a key factor in immune evasion and inhibition of T cell proliferation.^92^^,^^93^ In STING knockout mouse models of Lewis lung carcinoma (LLC), levels of IDO and myeloid-derived suppressor cells (MDSCs) in the TME were significantly reduced.^94^ Additionally, STING activation has been shown to promote the progression of cancers such as oral squamous cell carcinoma^95^ and HCC^96^ through upregulation of programmed cell death ligand 1 (PD-L1). Similarly, in endometrial cancer cells, mitochondrial DNA release and STING activation lead to increased PD-L1 expression, inducing T cell apoptosis.^4^^,^^97^ After intratumoral injection of STING agonists, PD-L1, IDO, and COX2 expression in the TME is elevated, impairing the anti-tumor efficacy of STING agonists.^98^ In tumors treated with STING-activating nanoparticles, there is an increase in B7 homolog 3 (B7-H3/CD276) expression, another immune checkpoint molecule linked to immune escape.^22^ The STING agonist ADU-S100 enhances the expression of T cell immunoglobulin domain and domain-3 (Tim-3) in type 2 conventional dendritic (cDC2) cells in both mice and humans, suppressing CD4^+^ T cell function and weakening CD4^+^ T cell-driven anti-tumor responses.^99^^,^^100^ In a mouse model of Renca lung metastasis, treatment with STING agonist-loaded lipid nanoparticles significantly increased lymphocyte activation gene-3 (LAG-3) expression in immune cells.^101^ These findings suggest that STING agonists can induce acquired resistance through upregulation of immune checkpoint molecules such as IDO, PD-L1, COX2, B7-H3, Tim-3, and LAG-3, aiding tumor immune evasion (Figure 4A).

#### Activation of secondary immunosuppressive cells and factors

Evaluating the therapeutic potential of STING agonists requires determining whether a T cell-enriched TME can be established to support cGAS-STING pathway activation, as a T cell-rich TME is associated with a positive response to STING agonists and improved prognosis.^34^ Additionally, BATF3-dependent DCs play a crucial role in STING-mediated anti-tumor immunity.^102^ Notably, STING is expressed in both immune and non-immune cells within the TME, including tumor cells and vascular endothelial cells.^13^^,^^103^ Activation of STING in different cell types within the TME can have contrasting effects: it may recruit immune-supportive cells to inhibit tumor growth and metastasis,^3^ but it can also attract immunosuppressive cells, promoting tumor progression.^104^ The activation of the cGAS-STING pathway can enhance the expression of immunosuppressive cells in the TME, including regulatory T cells (Tregs), regulatory B cells (Bregs), cancer-associated fibroblasts (CAFs), and MDSCs, contributing to an immunosuppressive TME that fosters cancer progression.^104^^,^^105^ In human papillomavirus (HPV)-positive tongue squamous cell carcinoma, STING activation promotes the production of immunosuppressive cytokines such as IL-10, facilitating Treg infiltration.^106^ In a pancreatic cancer mouse model, systemic administration of STING agonists induces Breg expansion through the STING-IRF3 axis, with Bregs secreting IL-35 and IL-10, which suppress NK cell activity and intratumoral infiltration.^107^ Arwert and co-workers have revealed that activation of the STING-IRF3 pathway in CAFs reduces the cytotoxic effects of oncolytic viruses on tumor cells, impacting the efficacy of oncolytic virotherapy. STING-dependent activation of MDSCs in MC38 mouse colorectal tumors after radiation exposure promoted macrophage M2 polarization and Treg proliferation.^108^^,^^109^ In neurofibromatosis type 1, STING activation recruits type 1 conventional dendritic cells and T cells that paradoxically promote tumor formation, particularly through CD8^+^ T cells.^110^ STING activation upregulates immunomodulatory factors, including IL-10 and IL-35, which inhibit anti-tumor immune cell activity and foster an immunosuppressive TME (Figure 4B).^4^^,^^106^^,^^107^ The immunomodulatory effect of STING activation remains unclear, and current STING agonists lack specificity for particular cell types within tumors.^55^

#### Inhibition of effector immune cells

Excessive STING activation may suppress T and B cell proliferation, reduce their infiltration, and promote apoptosis.^111^^,^^112^^,^^113^ Lemos et al. found that STING facilitates LLC growth by reducing CD8^+^ T cell infiltration while increasing MDSC infiltration within the TME.^94^ Furthermore, in tumors from *Sting1*^*S365A/S365A*^ mice, a significant number of T cells underwent STING-mediated, IFN-independent cell death, accelerating tumor progression and immune evasion.^114^ Larkin et al. found that DMXAA activated the stress pathways in T cells and upregulated the expression of pro-apoptotic genes (such as BAX), leading to T cell apoptosis. In a mixed bone marrow chimera model, STING-deficient conditions were associated with a greater number of Ki-67^+^ CD8^+^ memory T cells than controls.^111^ Mechanistically, T cells express high levels of STING and exhibit low type I IFN responses. While STING activation in DCs is essential for T cell recruitment and activation in tumors, STING activation in T cells triggers transcriptional changes dependent on NF-κB rather than IFN, ultimately leading to T cell apoptosis.^104^^,^^111^^,^^115^ Additionally, the LRRC8C transporter in T cells can transport cGAMP intracellularly, activating the STING-p53 pathway, inhibiting the influx of Ca^2+^ and the production of cytokines, and suppressing T cell-mediated adaptive immune responses.^116^^,^^117^ STING activation also diminishes B cell sensitivity to B cell receptor signaling and may induce apoptosis, thereby weakening antibody responses (Figure 4C).^112^^,^^118^^,^^119^

#### ER stress

Various genetic and environmental factors can impair the protein-folding and modification functions of the ER, resulting in the accumulation of misfolded proteins—a condition termed ER stress.^120^ ER stress is a known barrier to the anti-tumor efficacy of STING agonists.^121^ In T cells, the activation of STING, either directly or through Ca^2+^ flux, leads to a significant upregulation of genes involved in the unfolded protein response, particularly Bip/HSPA5 and GADD34.^112^ The accumulation of unfolded or misfolded proteins in the ER causes ER stress and impedes protein translation, enhancing immune evasion by tumor cells, allowing them to survive under stress and evade immune surveillance (Figure 4D).^122^^,^^123^^,^^124^

## Enhancing the efficacy of STING agonists through pharmacological modifications

### Increasing the metabolic stability of CDNs

**Figure 5:**
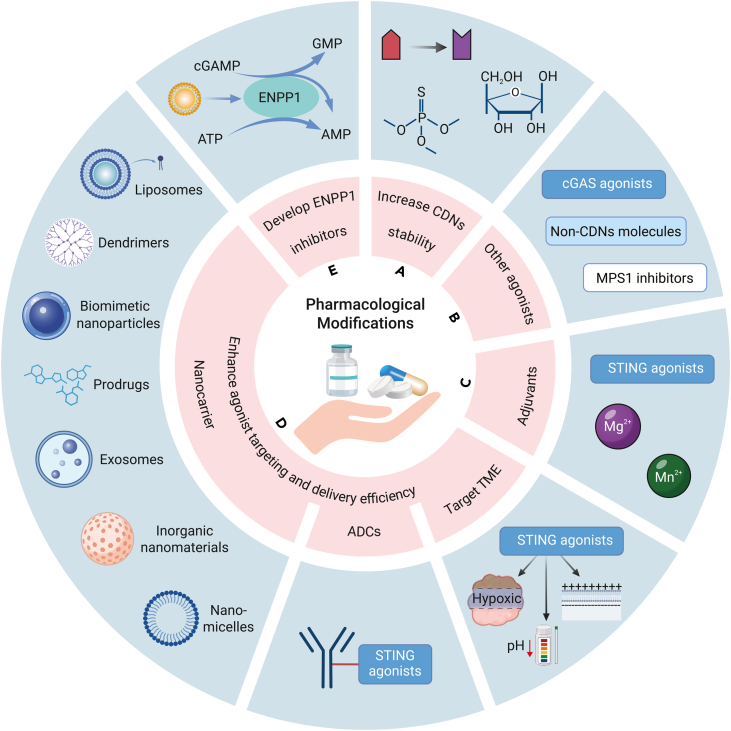
Enhancing the efficacy of STING agonists through pharmacological modifications Enhancing the efficacy of STING agonists involves addressing challenges in pharmacokinetics, tumor specificity, and immune activation. Strategies such as modifying CDNs, developing non-CDN agonists, utilizing Mn^2+^ adjuvants, improving targeted delivery via nanocarriers and ADCs, and developing ENPP1 inhibitors represent key approaches to optimize STING agonist therapies, making them more effective in cancer treatment.

Synthetic CDN analogs exhibit promising production potential^125^; however, their pharmacokinetic properties remain suboptimal due to limited membrane permeability and metabolic instability. To address these limitations, researchers have developed modified synthetic CDNs with enhanced degradation resistance by employing strategies such as nucleotide substitution, ribose replacement, and the incorporation of phosphothioate linkages.^126^^,^^127^^,^^128^^,^^129^ Nucleotide modifications and ribose substitutions have markedly improved type I IFN production, while phosphothioate linkage modifications confer CDN analogs with resistance to hydrolytic enzymes, including phosphodiesterases, nucleases, ENPP1, and snake venom phosphodiesterase (Figure 5A).^32^^,^^130^^,^^131^ For example, Leach et al. developed a positively charged multi-domain peptide hydrogel that enables sustained release of CDNs over 15 h, resulting in higher CDN concentrations at the injection site compared with free CDNs. This approach significantly inhibited tumor growth in an MOC2-E6E7 oral cancer mouse model, providing a potential strategy to advance CDNs for clinical use.^132^

### Development of non-CDN STING agonists and cGAS agonists

Second-generation STING agonists, primarily non-CDN small molecules, aim to overcome the pharmacokinetic limitations of nucleotide-based drugs. Notable examples include diABZI,^23^ MSA-2,^18^^,^^133^ and SR-717,^19^ each demonstrating unique advantages. The intravenously administered diABZI exhibits stronger innate immune activation than cGAMP,^23^ while MSA-2 and SR-717 are particularly promising due to their oral bioavailability.^134^ MSA-2, designed with protonated carboxylic groups, enhances cell membrane permeability in acidic TME, effectively promoting IFN-β secretion and inducing tumor regression in mouse models. When combined with anti-PD-1 therapy, MSA-2 significantly improves therapeutic efficacy with minimal adverse effects.^18^ However, systemic administration of MSA-2 raises concerns regarding off-target STING activation in other tissues, underscoring the necessity for further safety evaluations.

To better illustrate the advantages and limitations of CDN-based versus non-CDN STING agonists, Table 1 compares tumor selectivity, cytokine release profiles, and clinical efficacy indicators, such as plasma half-life and EC_50_ for hSTING variants. This comparative analysis provides a comprehensive framework for evaluating various drug classes and guiding the development of therapeutic strategies.

**Table 1:** Comparative study of CDN and non-CDN STING agonists in pharmacokinetics and efficacy

Types	STING agonists	Tumor selectivity	Half-life	EC_50_ (μM)	Cytokine release profiles
CDN STING agonists	2′3′-cGAMP	advanced/metastatic non-small cell lung cancer	2 min	4.1–26.3 (IFN-α/β); 22.2 -≥ 50 (TNF-α)	IFN-β, TNF-α, IL-6, CXCL10
	ADU-S100 (MIW815)	advanced or recurrent solid tumors	24 min	2.9	IFN-β, TNF-α, IL-6, CXCL10
	E7766	extensive-stage small cell lung cancer	NA	NA	transiently increased (peak <10 h) in IFN-α/β/γ, TNF-α, IL-6, CXCL10, CCL2, CCL4 and returned to baseline
	GSK532	advanced/metastatic solid tumors or lymphomas	NA	NA	IFN-β, TNF-α, IL-6, CXCL10, CCL2, CCL4
	JNJ-67544412 (JNJ-4412)	advanced/metastatic solid tumors or lymphomas	NA	NA	IFN-α, IFN-β, TNF-α, IL-6, CXCL10, CCL2
	BI-STING	advanced/metastatic solid tumors or lymphomas	NA	NA	IFN-β, TNF-α, IL-6, CXCL10, CCL2
	MK-1454	advanced/metastatic solid tumors or lymphomas	NA	NA	IFN-β, TNF-α, IL-6, CXCL10, CCL2
	TAK-676	metastatic/recurrent head and neck cancer	NA	0.3 ± 0.11 (HEK293T); 1.53 ± 0.45 (THP1-dual)	IFN-α, IFN-β, TNF-α, IL-6, CXCL10, CCL2
	BI-1387446	advanced solid cancers	NA	NA	IFN-β, TNF-α, IL-6, CXCL10, CCL2
	BMS-986301	refractory malignancies	NA	NA	IFN-β, TNF-α, IL-6, CXCL10, CCL2
	exoSTING	advanced solid tumors	tumor retention: retained; systemic exposure: none, well tolerated	NA	IFN-β, TNF-α, IL-6, CXCL10, CCL2
	SB 11285	solid tumors	NA	0.5	IFN-β, TNF-α, IL-6, CXCL10, CCL2
	IMSA-101	advanced solid tumors or lymphomas	NA	0.5	IL-18, IFN-β, TNF-α, IL-6, CXCL10, CCL2
non-CDN STING agonists	SNX281	advanced or metastatic solid tumors	2.33 h	NA	IFN-β, TNF-α, IL-6
	HG-381	advanced solid tumors and lymphoma	NA	NA	NA
	GSK3745417	metastatic or unresectable, recurrent head and neck squamous cell carcinoma	NA	NA	IFN-β, TNF-α, IL-6
	MK-2118	advanced or metastatic solid tumors	1.6–3.9 h	NA	IFN-γ, CXCL10, IL-6
	MSA-2	preclinical models of colorectal cancer (MC38), melanoma (B16.F10), and breast cancer (4T1)	MSA-2 (oral): 0.82 h; SAProsome-3: 4.61–5.95 h	8.3 (hSTING: WT); 24 (hSTING: HAQ)	IFN-β, TNF-α, IL-6
	SR-717	preclinical models of colorectal cancer	NA	2.1 (ISG-THP1); 2.2 (cGAS-KO ISG-THP1)	IFN-β
	diABZI	preclinical models of colorectal cancer (CT26)	1.4 h	0.035/0.055	IFN-β, IL-6, TNF-α, CXCL1

NA, not available.

Similarly, cGAS agonists are gaining attention for their ability to simulate endogenous cGAMP activation of STING.^135^ Techniques such as inducing mitotic stress or intracellular delivery of dsDNA provide promising avenues to emulate this natural activation process, offering new insights into STING pathway activation (Figure 5B).^136^^,^^137^

### Use of activation adjuvants

Metal ions such as Mn^2+^ and Mg^2+^ have shown potential as STING pathway activation adjuvants.^138^^,^^139^ Applied locally or delivered via drug platforms alongside STING agonists, these ions can enhance STING pathway activation.^55^^,^^140^^,^^141^^,^^142^^,^^143^^,^^144^^,^^145^^,^^146^ Mn^2+^, in particular, plays a critical role in cGAS-STING pathway activation and anti-tumor immunity.^134^^,^^147^^,^^148^^,^^149^ Studies reveal that Mn^2+^ deficiency in mice accelerates tumor growth and metastasis, accompanied by a notable reduction in tumor-infiltrating CD8^+^ T cells.^150^ Intracellularly, Mn^2+^ is sequestered within membrane-bound organelles, such as the Golgi apparatus and mitochondria, and released into the cytosol after viral infection. In the cytosol, Mn^2+^ has multiple roles in STING activation: it can independently activate monomeric cGAS without dsDNA, enhance cGAS-dsDNA binding through allosteric interactions, increase cGAS catalytic activity for higher cGAMP production, and possibly improve cGAMP affinity for STING. Additionally, Mn^2+^ can induce TBK1 phosphorylation independently of STING, promoting IFN-β production.^55^^,^^150^ Such properties make Mn^2+^ a noteworthy adjuvant for STING activation.^151^ For instance, Chen and co-workers reported that a Mn^2+^-cGAMP nanovaccine (Mn^2+^-cGAMP NV) significantly bolstered anti-tumor immunity in a mouse melanoma model, achieving superior control of primary and distal tumors when combined with anti-PD-L1 antibodies (Figure 5C).^21^

### Enhancing STING agonists targeting and delivery efficiency

Despite the potential of STING agonists for systemic administration, their lack of tumor specificity can lead to widespread inflammation. To achieve optimal therapeutic outcomes, STING agonists must specifically target tumor tissues, maximizing anti-tumor efficacy while minimizing adverse effects on normal tissues. This has driven the exploration of tumor-targeted, stable STING agonists and advanced drug delivery systems designed to optimize the pharmacological properties and compatibility of STING agonists with carrier materials, aiming for controlled, targeted delivery to tumors, enhanced intratumoral retention, and reduced off-target effects (Figure 5D).^55^^,^^152^^,^^153^^,^^154^^,^^155^^,^^156^^,^^157^

#### Targeting the TME via biochemical cues

The unique biochemical features of the TME, such as hypoxia, low pH, and altered membrane potential, offer avenues for targeted delivery of STING agonists.^18^^,^^158^ For instance, MSA-2 exhibits increased cell membrane permeability in the acidic TME, facilitating accumulation and STING activation within the tumor.^18^

#### Antibody-drug conjugates

Antibody-drug conjugates (ADCs) present significant potential for targeted delivery of STING agonists. By conjugating bioactive drugs with tumor-specific monoclonal antibodies, ADCs enable precise drug release at tumor sites, reducing toxicity to normal tissues.^45^ XMT-2056, an ADC combining diABZI with an anti-HER2 antibody, successfully induced robust anti-tumor immunity *in vivo* with 100-fold efficacy over diABZI alone. Although systemic cytokine levels showed only minor elevation,^159^^,^^160^ a phase I trial for XMT-2056 was halted after a grade 5 (fatal) severe adverse event, underscoring the importance of ensuring safety in innovative drug development.

#### Nanocarrier delivery strategies

Nanocarriers are emerging as promising delivery platforms, facilitating tumor-targeted delivery of STING agonists through mechanisms such as cellular phagocytosis.^149^^,^^161^^,^^162^^,^^163^^,^^164^^,^^165^^,^^166^^,^^167^ Their small size and large surface area allow efficient drug loading and extended circulation via the enhanced permeability and retention effect.^155^^,^^168^ Specifically modified nanocarriers can further enhance targeting, even crossing biological barriers such as the blood-brain barrier.^169^^,^^170^ For example, Dane et al. ingeniously designed lipid nanodisks (LNDs) carrying CDN-PEGylated lipids (LND-CDNs) to enhance membrane permeability and cellular uptake of CDNs. After intravenous injection, LND-CDNs effectively activated the STING pathway in tumors, triggering a robust immune response.^171^ Hanson et al. successfully delivered CDNs to draining lymph nodes using PEGylated lipid nanoparticles, achieving effects superior to those of a 30-fold dose of soluble CDNs.^172^ Huang et al. designed a tumor-targeted lipid-dendrimer-calcium phosphate nanocarrier, which not only exhibits excellent plasmid DNA delivery capabilities, but also effectively activates the STING pathway.^173^ A metal micellar nanovaccine (ONc-Mn-A-malF) composed of Mn, an STING agonist (ABZI), and naphthalocyanine (ONc)-coordinated nanoparticles (ONc-Mn-A), along with maleimide-modified Pluronic F127 (malF127) micelles, can effectively activate the STING pathway, promote the maturation of DC cells, and ultimately eliminate tumor cells through CD8^+^ T cells and NK cells.^174^ McAndrews et al. have also engineered exosomes (iExo^STINGa^) for the delivery of cGAMP to antigen-presenting cells (APCs), exhibiting superior tumor inhibition compared with single-agent STING agonists.^175^ Additional examples include: nanoparticle-cGAMP, which enhances the cytosolic release of cGAMP^176^; nanoparticle PC7A NP, which elicits a robust cytotoxic T cell response while minimizing systemic cytokine expression^177^; ZnS@BSA (bovine serum albumin), which releases zinc ions in the acidic TME to enhance cGAS/STING signaling significantly; and the cGAS-STING nanoagonist (BSA-Man@Mn^2+^-Ft@Lap), which enhances the tumor-specific T cell-mediated immune response against poorly immunogenic solid tumors *in vivo*.^178^

Key objectives in selecting nanocarrier-based delivery systems for STING agonists include maximizing tumor-specific targeting, extending half-life, and ensuring efficient cellular uptake and cytoplasmic delivery, ultimately achieving effective STING activation at the ER with minimal off-target effects.^34^^,^^179^^,^^180^

### Development of ENPP1 inhibitors

To counteract CDN instability, ENPP1 inhibitors have been developed to locally inhibit ENPP1 within tumors, effectively increasing extracellular cGAMP concentration while reducing immunosuppressive adenosine production.^46^^,^^181^ ENPP1 inhibitors are generally divided into nucleotide-based and non-nucleotide-based types.^182^ The former, although relatively easy to synthesize, exhibit limited selectivity, low oral bioavailability, and risk of off-target effects due to their structural similarity to natural substrates.^183^ In contrast, non-nucleotide ENPP1 inhibitors are being explored, including polysulfonates, polymetallic compounds, polysaccharides, and heterocyclic compounds. Although early-stage research on non-nucleotide ENPP1 inhibitors faces challenges, these compounds offer a promising new approach to enhancing anti-tumor immunity in cancer therapy (Figure 5E).^183^

## Improving treatment outcomes: Strategies for dosing and development of STING agonists

### Exploring molecular mechanisms underlying STING activation

**Figure 6:**
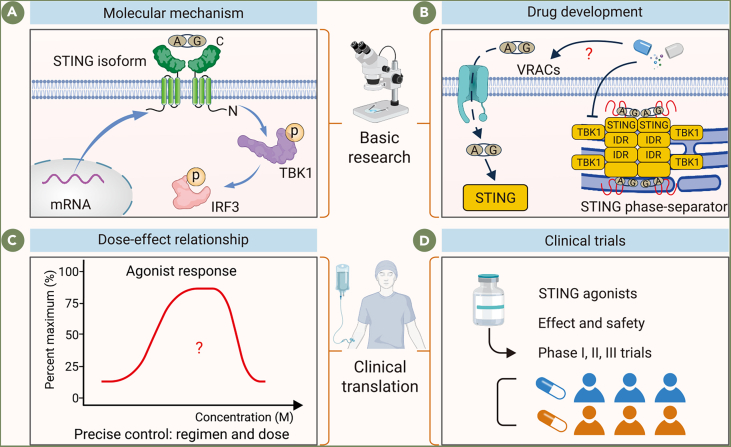
Strategic framework for optimizing STING-based immunotherapy (A) Molecular mechanism: Schematic representation of pmSTING-mediated immune activation. The distinct topology and activation mechanism of pmSTING highlight its potential as an alternative immunotherapeutic target. (B) Drug development: Various strategies including phase separation regulation and VRAC-mediated delivery are under investigation to enhance STING agonist performance. (C) Dose-effect relationship: A bell-shaped dose–response curve suggests that both insufficient and excessive dosing can compromise therapeutic efficacy, underscoring the need for precisely optimized dosing regimens. (D) Clinical trials: Expanding high-quality trial evidence is crucial to support systematic evaluation and guide rational clinical application. VRACs, volume-regulated anion channels.

The Zheng team recently identified a novel plasma membrane-bound STING isoform, termed “plasmatic membrane STING (pmSTING).”^30^^,^^184^ Unlike the classical endoplasmic reticulum-bound STING (erSTING), pmSTING’s C terminus is extracellular, allowing it to directly sense extracellular cGAMP, inducing TBK1 and IRF3 phosphorylation and triggering an immune response. This discovery highlights pmSTING’s potential evolutionary conservation across species. Traditionally, research has focused on erSTING, with minimal exploration into anti-tumor therapies targeting pmSTING. This shift of focus could aid in reducing drug dosages, enhancing therapeutic indices, and minimizing systemic toxicity and side effects (Figure 6A).

### Developing STING phase separation inhibitors and VRAC channel agonists

At elevated cGAMP concentrations, STING undergoes liquid-liquid phase separation, forming STING phase separators that sequester STING and TBK1, limiting type I IFN production and preventing immune overactivation.^185^^,^^186^^,^^187^ Microtubule-disrupting agents inhibit this gel-like transition of STING condensates, providing a novel approach to enhance STING activation.^185^ Apart from conventional STING agonists, other drugs, such as VRAC channel agonists, show promise in augmenting STING signaling, although further exploration in cancer immunotherapy remains warranted (Figure 6B).^55^

### Optimizing dosage and administration of STING agonists

To maximize the anti-tumor efficacy of STING agonists, it is essential to monitor their half-life within the TME, allowing precise control over dosing and administration schedules. Enhancements to existing nanoparticle delivery systems may also be beneficial, particularly if these carriers can deliver STING agonists alongside other agents, leveraging synergistic anti-tumor effects. Simplifying preparation steps would further improve clinical feasibility. However, it is critical to note that prolonged exposure to high STING agonist concentrations may lead to T cell apoptosis, counteracting the intended immune activation.^112^^,^^115^ Therefore, comprehensive safety assessments must precede clinical development, ensuring therapeutic efficacy while minimizing immunotoxicity to protect patient safety (Figure 6C). It is important to note that optimizing the dosage of STING agonists and innovating administration methods not only enhances the anti-tumor efficacy but also reduces the risk of CRS associated with the systemic application of these agents. Furthermore, monitoring CRS biomarkers, such as IL-10, IL-6, and ferritin, can aid in the detection of CRS occurrence.^188^

### Advancing clinical trials for STING agonists numerous

STING agonists are undergoing clinical trials, offering valuable insights into optimal dosage, delivery methods, and combination therapies. A clinical study demonstrated a higher objective response rate (ORR) of 16.2% in first-line immunotherapy patients, compared with 7.2% in those with prior immunotherapy, highlighting the advantage of administering STING agonists before PD-1 inhibitors to more effectively activate the immune system. Notably, a patient with triple-negative breast cancer, who had not previously received PD-1 treatment, was treated with a combination of 200 μg MIW815 and spartalizumab. This resulted in complete remission after five cycles of treatment, suggesting that patients who have not previously received immunotherapy may particularly benefit from combining STING agonists with PD-1 inhibitors.^189^

The studies are accelerating the development of next-generation agents with improved targeting and reduced side effects.^134^ Despite the diverse range of STING agonists in clinical trials, a shortage of published clinical data hinders a comprehensive understanding of their efficacy and safety profiles. Expanded clinical trial data would support a more systematic evaluation of their therapeutic value and guide future clinical applications to achieve safer, more effective treatments (Figure 6D).

## Tailored approaches: Strategies and research directions for enhancing STING agonists efficacy

### New strategies for CIN-high tumor patients unsuitable for STING agonists

**Figure 7:**
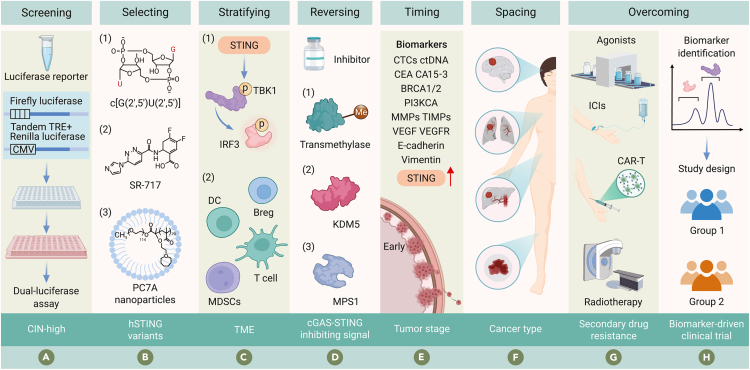
Tailored approaches: Strategies and research directions for enhancing STING agonists efficacy Through personalized STING agonist therapy, combined with tumor characteristics and microenvironmental factors, we can maximize anti-tumor efficacy. (A) New strategies for CIN-high tumor patients unsuitable for STING agonists. (B) Selecting STING agonists based on hSTING variants to improve efficacy. (C) Stratified treatment based on tumor and microenvironment biomarkers. (D) Reversing inhibition of tumor cell cGAS-STING signaling. (E) Optimizing STING agonists timing based on metastasis stages. (F) Selecting approaches based on the spacing of cancer type and STING expression. (G) Overcoming secondary resistance to STING agonists with combination therapies. (H) Design clinical trials based on biomarkers.

The stratified use of STING agonists and inhibitors is crucial due to the rapid adaptation of downstream STING pathways that enable immune evasion. Patient stratification considering tumor stage, gene expression, and CIN scores is essential. Identifying patients who would benefit from STING activation versus those who may require STING inhibition due to TME-induced stress will optimize therapeutic efficacy.^108^ Additionally, monitoring key molecules in both classic and non-classical STING pathways is vital during STING agonist treatment. activation of IRF3 and NF-κB should be closely monitored, with tools like luminescence reporter systems, such as ISRE-luciferase, reflecting IRF3 activity, while dual-reporter systems (THP-1-dual) enable IRF3 and NF-κB activation assessments (Figure 7A).^128^^,^^190^

### Selecting STING agonists based on hSTING variants to improve efficacy

Selecting STING agonists based on hSTING mutations involves genetic sequencing to identify specific hSTING mutations in cancer patients, enabling selection of STING agonists with high activity against these variants. However, the diversity and uneven distribution of hSTING gene variants present a challenge in this selection. To address this, developing broad-spectrum STING agonists, such as the novel cyclic dinucleotide analog, c[G(2′,5ʹ)U(2′,5ʹ)], which activates all known hSTING variants with efficacy comparable with cGAMP (Figure 7B(1)).^191^ Although its *in vivo* anti-tumor effects remain unverified, it serves as a critical reference for designing universal STING agonists. SR-717’s binding mode, unaffected by STING allelic differences, further supports the exploration of such agents in diverse patient populations (Figure 7B(2)).^19^ In addition to broad-spectrum agonists, designing certain STING agonists selectively modulates STING activity. For example, acid-responsive nanoparticles (PC7A) bind and activate STING noncompetitively, effectively targeting cGAMP-insensitive hSTING variants and delivering rapid and sustained biological effects (Figure 7B(3)).^177^^,^^192^^,^^193^

### Stratified treatment based on tumor and microenvironment biomarkers

Biomarkers predicting STING agonist efficacy are crucial for patient selection. Key molecules in the STING pathway, such as pTBK1 and pIRF3, are essential for signal transduction before the nuclear translocation of pIRF3 and can serve as biomarkers to predict the efficacy of STING agonists. In conjunction with T cell and BATF3-lineage DC analyses in the TME, these markers enable more precise patient selection (Figure 7C(1)). Furthermore, exploring cell-type-specific responses to STING agonists is important for developing more effective, cell-targeted therapies. Investigating the effects and influencing factors of STING agonists responses across different TME cell types could inform the development of cell-targeted STING agonists (Figure 7C(2)).

### Reversing the inhibition of tumor cell cGAS-STING signaling

Combination with DNA methyltransferase inhibitors (DNMTis) can enhance STING agonists efficacy by inhibiting DNA methyltransferase activity, reprogramming epigenetic profiles to restore STING signaling and improve tumor immunogenicity (Figure 7D(1)).^194^ Similarly, combining with KDM5 inhibitors has been shown to restore STING protein levels in breast cancer cell lines, supporting IFN responses akin to DNMTis (Figure 7D(2)).^84^ Furthermore, the combination with MPS1 inhibitors, such as BAY-1217389, induces CIN and micronuclei formation, activating STING signaling. This reactivation restores T cell infiltration in KRAS-LKB1 mutant lung cancers, overcoming STING silencing due to mitochondrial dysfunction (Figure 7D(3)).^88^

### Optimizing STING agonist timing based on metastasis stages

Researchers aim to exploit STING pathway activation to either eliminate cancer cells before metastasis or maintain them in a dormant state.^90^ In the intrahepatic cholangiocarcinoma mouse model, MSA-2 treatment showed specific inhibitory effects on early-stage tumors, but not on late-stage cancer.^195^ Although STING agonists are currently being evaluated in late-stage cancer patients, early-stage metastatic treatment may prove more effective due to higher STING expression and the absence of fully established immune-evasive TMEs (Figure 7E).

### Selecting approaches based on the spacing of cancer type and STING expression

Accounting for variability among cancer types is crucial in clinical practice. This approach involves stratifying patients based on STING and cGAS expression levels in the TME and CIN scores, enabling tailored STING agonist therapy while avoiding unnecessary treatments for insensitive tumor types (Figure 7F).

### Overcoming secondary resistance to STING agonists with combination therapies

To counteract secondary resistance mechanisms, such as immune checkpoint molecule upregulation, activation of immunosuppressive cells, and reduced immune effector cell activity, STING agonists are increasingly being combined with various therapies. These combinations enhance therapeutic efficacy across surgery, chemotherapy, radiotherapy, photodynamic therapy (PDT), CAR-T, and immune checkpoint inhibitors (ICIs).^108^^,^^169^^,^^196^^,^^197^ For example, Shi et al. showed that the combination of the STING agonist diABZI and the IDO inhibitor 1-MT significantly promotes the recruitment of CD8^+^ T cells and DCs while concurrently reducing the infiltration of MDSCs, resulting in notable anti-tumor effects.^93^ Lemos et al. found that the combination of STING agonist cyclic diadenyl monophosphate (CDA) and IDO1 inhibitor lindrostat significantly enhanced anti-tumor efficacy in the mouse LLC model, thereby prolonging the survival of mice.^98^ Song et al. proved that the combination of STING agonists and Toll-like receptor 2 agonists reduced the expression of PD-L1 in tumor monocytes, reshaped the TME, and enhanced anti-tumor immune responses.^198^ In a head and neck squamous cell carcinoma model, local delivery of STING agonists reduced recurrence risk post-surgical resection.^199^ Additionally, 5-FU combined with cGAMP enhanced anti-tumor effects while minimizing gastrointestinal side effects in murine models.^200^ Notably, the combined treatment of electroacupuncture and anti-PD-1 therapy also strengthens anti-tumor immune responses in microsatellite-stable colorectal cancer by activating the STING pathway (Figure 7G).^167^

### Design of clinical trials based on biomarkers

Advancing the precision of STING agonist therapy necessitates the incorporation of biomarker-guided clinical trial design. By utilizing biomarkers that indicate the activation status of the STING pathway, such as pTBK1 and pIRF3, researchers can more accurately predict which patients are likely to respond positively to STING agonists. The integration of these biomarkers into clinical trial design facilitates minimizing treatment-related adverse effects while maximizing therapeutic efficacy. As the field progresses, collaboration between biomarker identification and clinical trial design will be essential in unlocking the full potential of STING agonists in cancer immunotherapy, leading to more effective and safer treatment regimens (Figure 7H).

## STING-related drugs in clinical development

### DMXAA

**Figure 8:**
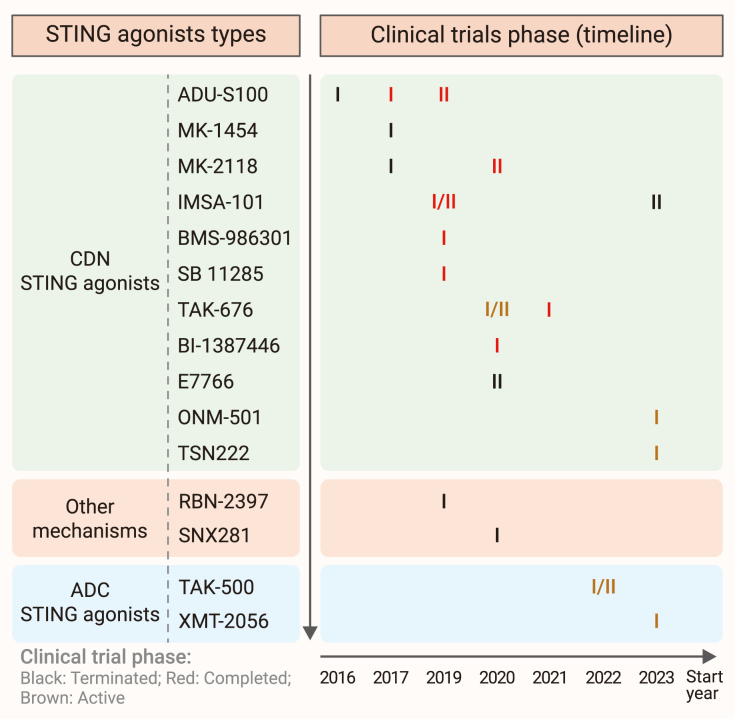
Clinical development of STING agonists: therapeutic strategies and trial outcomes A timeline summarizes the clinical development of various STING agonists, with each compound positioned according to its respective clinical trial phases. Color coding is used to indicate the current trial status (e.g., terminated, completed, or ongoing), providing a clear overview of therapeutic strategies and trial outcomes.

The mSTING agonist DMXAA is the leading molecule to enter clinical trials with several phase I and II clinical trials confirming its safety and activity profile in a few solid tumors (Table 2). A phase I clinical trial was conducted in Japanese patients to assess the safety, tolerability, and preliminary efficacy of ASA404 in combination with docetaxel for the treatment of advanced or recurrent solid tumors (NCT01285453).^201^ The study enrolled nine patients, and the results showed that the combination of ASA404 and docetaxel had an acceptable tolerability profile, but the efficacy was limited, with one patient (11.1%) achieving a partial response (PR) and five patients (55.6%) achieving stable disease (SD). In a phase II clinical study (NCT01057342), the addition of DMXAA showed unexpected effects in advanced non-small cell lung cancer patients compared with carboplatin and paclitaxel alone.^202^ Also, in phase III clinical trials (NCT00662597), DMXAA failed to show frontline efficacy in advanced non-small cell lung cancer (NSCLC) for its species-specific differences, which resulted in its termination.^203^ This therapeutic failure highlights the critical importance of STING protein polymorphisms and underscores the necessity of thoroughly understanding drug-target interactions in humans. Recently, Temizoz and co-workers suggested that the efficacy of DMXAA in early clinical results might contribute to its beneficial antagonism of STING-mediated immune responses, which deserves further exploration.^28^^,^^204^ Nonetheless, the clinical exploration of STING agonists stepped into the development of hSTING activators (Figure 8).

**Table 2:** Completed, terminated, or recruiting clinical trials of STING-related drugs

NCT number	Treatment regimens	Cancer type	Administration of STING agonist	Phase	Status	Start year	Enrollment, participants (until today)
NCT00662597^a^	ASA404 (DMXAA) + paclitaxel + carboplatin	advanced/metastatic non-small cell lung cancer	i.v.	III	terminated	2008	1,299
NCT01285453^a^	ASA404 (DMXAA)^201^	advanced or recurrent solid tumors	i.v.	Ⅰ	completed	2009	9
NCT01057342^a^	ASA404 (DMXAA) + paclitaxel + carboplatin^202^	extensive-stage small cell lung cancer	i.v.	II	completed	2010	17
NCT02675439^a^	ADU-S100 + ipilimumab^205^	advanced/metastatic solid tumors or lymphomas	i.t.	Ⅰ	terminated	2016	47
NCT03010176^a^	MK1454 + pembrolizumab^205^	advanced/metastatic solid tumors or lymphomas	i.t.	Ⅰ	completed	2017	156
NCT03172936^a^	ADU-S100+PD-1 checkpoint inhibitor (PDR001)^205^	advanced/metastatic solid tumors or lymphomas	i.t.	Ⅰ	terminated	2017	106
NCT03249792^a^	MK-2118 + pembrolizumab	advanced/metastatic solid tumors or lymphomas	i.t./s.c.	Ⅰ	terminated	2017	140
NCT03422510^a^	CXA-10	primary focal segmental glomerulosclerosis	oral	II	completed	2018	33
NCT03449524^a^	CXA-10	pulmonary arterial hypertension	oral	II	terminated	2018	69
NCT04125745^a^	CXA-10	pulmonary arterial hypertension	oral	II	terminated	2019	1
NCT03937141^a^	ADU-S100 + pembrolizumab^205^	metastatic/recurrent head and neck cancer	i.t.	II	terminated	2019	16
NCT03956680^a^	BMS-986301 + nivolumab + ipilimumab	advanced solid cancers	i.m./i.t./i.v.	Ⅰ	completed	2019	54
NCT04020185^a^	IMSA101 + ICIs/immuno-oncology therapy	refractory malignancies	i.t.	Ⅰ/II	completed	2019	40
NCT04096638^a^	SB 11285 + atezolizumab	advanced solid tumors	i.v.	Ⅰ	completed	2019	61
NCT04053673^a^	RBN-2397	solid tumors	p.o.	Ⅰ	unknown status	2019	130
NCT04096638^a^	SB 11285 + atezolizumab	advanced solid tumors	i.v.	Ⅰ	completed	2019	61
NCT04144140^a^	E7766	advanced solid tumors or lymphomas	i.t.	Ⅰ	terminated	2020	24
NCT04420884^a^	TAK-676 + pembrolizumab^209^	advanced or metastatic solid tumors	i.v.gtt	Ⅰ/II	recruiting	2020	374
NCT04609579^a^	SNX281 + pembrolizumab	advanced solid tumors and lymphoma	i.v.gtt	Ⅰ	terminated	2020	27
NCT04220866^a^	MK-1454 + pembrolizumab^125^	metastatic or unresectable, recurrent head and neck squamous cell carcinoma	i.t.	II	completed	2020	18
NCT04147234^a^	BI-1387446 + ezabenlimab (BI-754091)	advanced or metastatic solid tumors	i.t.	Ⅰ	completed	2020	39
NCT04592484^a^	CDK-002 (exoSTING)	advanced/metastatic, recurrent, injectable solid tumors	i.t.	Ⅰ/II	completed	2020	27
NCT04053543^a^	CXA-10	pulmonary arterial hypertension	Oral	II	terminated	2020	33
NCT04879849^a^	TAK-676 + pembrolizumab after radiation^209^	non-small cell lung cancer, triple-negative breast cancer, or squamous cell carcinoma of the head and neck that has progressed on checkpoint inhibitors	i.v.gtt	Ⅰ	completed	2021	34
NCT04998422,^a^	HG381	advanced solid tumors	i.v.	Ⅰ	recruiting	2021	57
NCT05070247^a^	TAK-500 + pembrolizumab	locally advanced or metastatic solid tumors	i.v.gtt	Ⅰ/II	recruiting	2022	313
NCT05424380^a^	GSK3745417	relapsed/refractory AML or HR-MDS	i.v.	Ⅰ	terminated	2022	18
NCT05842785^a^	TSN222	advanced solid tumors or lymphomas	i.t.	Ⅰ/II	recruiting	2023	162
NCT06022029^a^	ONM-501 + cemiplimab	advanced solid tumors and lymphomas	i.t.	Ⅰ	recruiting	2023	168
NCT05514717^a^	XMT-2056	advanced/recurrent solid tumors that express HER2	i.v.	Ⅰ	recruiting	2023	162
NCT05846659^a^	IMSA101 + PULSAR-ICIs	oligoprogressive solid tumor malignancies	i.t.	II	terminated	2023	16

i.v., intravenous injection; i.t., intratumoral injection; i.m., intramuscular injection; s.c., subcutaneous injection; p.o., per os; i.v.gtt, intravenous infusion.

a https://clinicaltrials.gov

### CDN STING agonists

Developed by Aduro Biotech, ADU-S100 has aggressively entered several clinical trials in collaboration with Novartis in the past decade. Although exhibiting exciting efficacy in preclinical models, the compound showed limited clinical activity either as monotherapy (NCT02675439) or combined with ICIs (NCT03172936 and NCT03937141).^189^^,^^205^ Accordingly, in the phase I trial of ADU-S100 monotherapy (NCT02675439) for advanced/metastatic solid tumors or lymphomas, the agent was well tolerated upon intratumoral administration without dose-limiting toxicities.^27^ However, few patients benefited from the treatment, with only 2.1% of patients (*n* = 1/47) achieving PRs and 38% of patients (*n* = 18/47) achieving SD. Afterward, ADU-S100 was explored together with the PD-1 inhibitor spartalizumab in a phase Ib study (NCT03172936), eliciting slightly better responses compared with monotherapy. The interim data showed that 17 of the 23 enrolled triple-negative breast cancer (TNBC) patients were evaluable for efficacy, among whom 1 (4.3%) had achieved a complete response and 3 (13%) had showed PRs.^189^ Thirty-five of the 38 melanoma patients were radiologically evaluable for efficacy, among whom 3 (7.9%) attained PRs and 12 (31.6%) achieved SD. Similarly, the phase II clinical trial combining ADU-S100 with the anti-CTLA4 antibody pembrolizumab (NCT03937141) showed weak clinical data and has been halted as a result of non-cooperation between Aduro Biotech and Novartis.^205^ Up to now, all ADU-S100 clinical trials have been terminated based on the disappointing clinical efficacy (Table 2). Several factors may contribute to this outcome. Firstly, the pharmacokinetic properties of ADU-S100 were suboptimal, characterized by rapid absorption and a short half-life after intratumoral administration,^206^ which likely limited the drug’s ability to maintain effective concentrations at the target site. Secondly, the route of administration and dosing schedule may not have been optimized to maximize the drug’s therapeutic potential. Additionally, the induction of immunosuppressive cells, such as MDSCs,^207^ may have counteracted the intended immune-activating effects of the STING agonist. These findings highlight the importance of thoroughly evaluating the pharmacokinetics and pharmacodynamics of a drug in humans and optimizing the treatment protocol accordingly.

The drug candidate ulevostinag (MK-1454) is being developed by Merck and tested in two human clinical trials for cancer immunotherapy (Table 2).^125^ In the phase I clinical trial of MK1454 alone or in combination with pembrolizumab (NCT03010176), MK-1454 monotherapy showed zero efficacy and the combination treatment showed ORR up to 16.1% in patients with cutaneous or subcutaneous lesions.^205^ All patients enrolled experienced treatment-related adverse events (TRAEs) and 10.6% of patients (*n* = 18/170) discontinued the trial due to severe TRAEs. This suggests that the combination treatment, although showing some promise, may not have been optimized for patient safety and tolerability. Future trials should focus on refining the dosing regimen and identifying patient subgroups who are more likely to benefit from the treatment while minimizing adverse effects. In its promising phase II study in combination with pembrolizumab (NCT04220866), the combination group attained an ORR of 50% (*n* = 4/8) as the first-line treatment of metastatic or unresectable, recurrent head and neck squamous cell carcinoma, compared with 10% (*n* = 1/10) for pembrolizumab alone.^125^

Created by ImmuneSensor Therapeutics, another small-molecule cGAMP analog IMSA-101 demonstrated strong immunostimulatory activities and anti-tumor efficacy in preclinical studies (Table 2). The phase IIa study of IMSA-101 alone or in combination with an ICI (NCT04020185) has recently completed, with the phase I study already having proven its safety and tolerability. While no response evaluation criteria in solid tumors (RECIST)-defined response was observed in the monotherapy cohort, significant tumor regression was observed in both injected and non-injected lesions. In the combination cohort, one patient (5.6%) with refractory uveal melanoma achieved a durable PR (with a 66% reduction in tumor size). Now IMSA-101 has been aggressively explored in phase II studies combined with PULSAR radiotherapy and ICI immunotherapy in patients with metastatic malignancies (NCT05846659 and NCT06601296).^208^ So far, the exact chemical structure of IMSA-101 has not been disclosed yet.

Designed by Takeda Pharmaceutical Company, dazostinag (TAK-676) is a synthetic novel CDN STING stimulator exhibiting intense STING-dependent anti-tumor effects upon intravenous administration in syngeneic murine tumor models, stimulating cytokine responses and robust activation of immune cells in the TME. Preclinical studies have identified TAK-676 as a promising therapeutic candidate and several clinical trials have been launched to evaluate its clinical efficacy (Table 2). The phase I study of TAK-676 plus pembrolizumab after radiation therapy (NCT04879849) has just completed recently, awaiting publication of results, with the expectation of boosting the immune response to resist ICI drug resistance via enhanced STING-IFN signaling.^209^ Another ongoing phase I and II study of TAK-676 alone or in combination with pembrolizumab (NCT04420884) is currently under recruiting status in patients with advanced solid tumors and is estimated to be completed in 2026.^209^ Now a phase I clinical trial has been registered in China (CTR20234291) to access the safety, tolerability, pharmacokinetics, and pharmacodynamics of TAK-676 alone or in combination with pembrolizumab, but which has not yet begun recruiting.

MK-2118 is another CDN analog developed by Merck with its structure remaining unknown (Table 2). In 2017, its phase I study was launched to assess the safety, tolerability and recommended phase 2 dose (RP2D) of MK-2118 alone or in combination with pembrolizumab, with MK-2118 administrated either intratumorally or subcutaneously (NCT03249792).^210^ Posted results showed that in patients with advanced/metastatic solid tumors or lymphomas, 4.9% patients (*n* = 7/140) are reported to have dose-limiting toxicities (DLT) and 11 patients reported TRAE discontinuation. Now the study has been terminated because of business reasons. This indicates that future trials must optimize dosage and administration frequency based on the results of pharmacokinetic and pharmacodynamic studies to enhance the treatment’s safety and efficacy.

BMS-986301 is a next-generation CDN STING agonist initially designed by IFM Therapeutics, and was acquired by Bristol-Myers Squibb afterward (Table 2).^45^ It led to dramatic tumor regressions in mouse colorectal tumor models either alone or combined with ICIs, while it induced immunological memory in CT26 murine models. Recently, researchers have found that intramuscular injection of BMS-986301 can achieve equivalent effector T cell activation and anti-tumor efficacy in PDAC mouse models compared with intratumoral administration, while effectively attenuating T cell exhaustion and immunosuppressive signals. The same abscopal anti-tumor activity was also observed in the mouse pancreatic and liver orthotopic model, further supporting the clinical development of systemic administration of the agent.^45^ Now, the phase I study of BMS-986301 alone or in combination with nivolumab and ipilimumab (NCT03956680) has just completed, the aim of which is to evaluate the safety, tolerability, and DLTs of multiple administration routes (i.m., i.t., or i.v.) of BMS-986301.

BI-1387446 (also known as BI-STING), is an optimized CDN STING agonist designed by Boehringer Ingelheim, which is reported to have similarities to the natural ligand of STING protein (Table 2).^211^ It exhibits superior activity and selectivity toward all major hSTING genotypes and induces lasting tumor inhibition and immunological memory even in low doses upon intratumoral administration. The phase I, first in-human study of BI-1387446 alone or in combination with ezabenlimab (BI-754091) has just completed in patients with solid tumors, but awaiting publication of results (NCT04147234).

E7766, a novel macrocycle-bridged STING agonist, is a pan-genotypic STING agonist showing potent binding affinity toward four major hSTING variants. In murine CT26 liver metastasis tumor models, intratumoral administration of E7766 exhibited high efficacy and long-lasting immunological memory response (Table 2).^212^ Moreover, in Bacillus Calmette-Guérin (BCG)-insensitive non-muscle invasive bladder cancer (NMIBC) models resistant to anti-PD-1 agents, intravesical instillation of E7766 showed great anti-tumoral activity accompanied with robust activation of the STING-IFN pathway and induction of downstream cytokines.^213^ These promising results had promoted the phase I study of E7766 as a single agent administered intravesically in BCG-unresponsive NMIBC patients (NCT04109092), but the study was withdrawn without enrollment of participants. Another phase I study of E7766 alone in participants with advanced solid tumors or lymphomas upon intratumoral administration (NCT04144140) was terminated because of the sponsor’s strategic decision, which was unrelated to safety; therefore, no participants were enrolled in the dose expansion part. According to the partial results released from the dose escalation part, there were 18.2% (*n* = 2/11) and 16.7% (*n* = 1/6) DLTs in the 600 and 780 μg groups, respectively. All participants experienced at least one TRAE and eight serious adverse events were reported. Clinical trials of E7766 must further investigate the optimal dosage and administration schedule to enhance treatment safety and efficacy.

Also, CDN STING agonists such as SB 11285 (NCT04096638), DN-015089 (CTR20212462), TSN222 (NCT05842785), and ONM-501 (NCT06022029) have also entered clinical trials (Table 2) in in patients with advanced solid tumors (Figure 8).^214^^,^^215^

### Engineered bacteria vector

A bacterial-based STING activator, SYNB1891, is an engineered strain of *Escherichia coli* Nissle, which is able to convert ATP to CDA under conditions of hypoxia to activate STING.^216^ Utilizing the active phagocytosis of bacteria by APCs, SYNB-1891 precisely reduces “off-target” effects and potently drives the activation of the STING-IFN pathway in phagocytic APCs within the TME, while activating complementary innate immune pathways through pattern-recognition receptors as well. Preclinical studies have demonstrated its efficacious anti-tumor activity and ability to establish immunological memory in B16.F10 tumor-bearing mice. Moreover, *in vitro* studies proved that this novel agonist can trigger the activation of multiple hSTING alleles. The convincing preliminary results put forth the development of SYNB1891 in a phase I clinical trial (NCT04167137) in refractory advanced solid tumors or lymphoma (Table 2).^56^ According to the released results, SYNB1891 alone or in combination with atezolizumab exhibited safety and tolerability upon intratumoral administration with the observation of dose-related induction of serum cytokines (i.e., TNF-α, IL-6, IFN-γ, and IL1RA). TRAEs were observed in all patients, and only 1 DLT out of 32 patients was reported. Thirty-six percent of patients (*n* = 9/25) achieved SD, 4 of whom are refractory to previous anti-PD-1/L1 treatment, while the other 64% evaluable participants experienced PD. Although 11 out of 32 patients reported to have serious adverse events, no infectious EcN-associated toxicities were witnessed. However, the change in the study sponsor’s corporate focus away from oncology has led to the discontinuation of its first-in-human study, thus the trial has been terminated prematurely by the sponsor before enrollment completion (Figure 8).

### ADC STING agonists

ADCs have been extensively explored for targeted STING agonist delivery upon systemic administration. Taking advantage of the antibody against tumor-specific antigens, STING agonist ADCs precisely direct STING agonists into cancer cells with reduced toxicity and off-target effects. Compared with unconjugated (free) agonists, STING agonist ADCs induced 100-fold higher levels of cytokines and IFN as well as enhanced tumor cell death during *in vitro* assays. Multiple preclinical murine studies have also proved its target-dependent anti-tumor immunity with significant increase in tumor-localized inflammatory cytokines and immune cell infiltration, yet the systemic cytokine levels surprisingly remained low (Figure 8).^217^

XMT-2056, an optimized STING agonist ADC by Mersana Therapeutics, targets a novel HER2 epitope based on the specifically tailored Immunosynthen platform. Initial data demonstrated its *in vivo* anti-tumor activity either alone or in combination with other HER2-targeting drugs and ICIs. Also, XMT-2056 exhibited favorable pharmacokinetics and tolerability in non-human primates even at high doses. Additionally, XMT-2056 also showed antibody-dependent cell-mediated cytotoxicity function in synergy with STING activation to enhance cancer cell-killing efficacy (Table 2).^218^ Based on these preclinical results, Mersana Therapeutics launched a phase I clinical trial of XMT-2056 alone in HER2-positive recurrent or metastatic solid tumors (NCT05514717).^219^ Tragically, a grade 5 (fatal) serious adverse event occurred in its dose escalation part in March 2023, with the death of the second patient who had been enrolled at the initial dose level, which led to the transient pause of the study. The underlying cause has not yet been disclosed, which arouses attention to the safety and biological balance of targeting STING with ADC agonists. In late 2023, the US Food and Drug Administration reached the agreement of the continuation of the trial, with a lower starting dose. Now the study is actively recruiting and is estimated to be completed in 2027.

Another immune-stimulating antibody conjugate (ISAC) STING agonist, TAK-500, has been developed by Takeda based on TAK-676 and an IgG1 anti-cysteine-cysteine chemokine receptor 2 (CCR2) antibody. The novel ISAC is designed to overcome the tumor resistance to ICIs by targeted delivery of STING agonists to tumor-infiltrating CCR2-expressing myeloid cells, including tumor-associated macrophages (TAMs) (Table 2).^220^ Preclinical mouse models have validated its effect in boosting the anti-tumor immunity by inducing the accumulation and activation of CD8^+^ effector T cells in the TME, thus resulting in enhanced survival. TAK-500 is demonstrated to function via three aspects: activation of IFN signaling, reprogramming of CCR2-expressing myeloid cells to an inflammatory phenotype, and blockade of suppressive TAM recruitment. The phase I study of TAK-500 with or without pembrolizumab in adults with select locally advanced or metastatic solid tumors (NCT05070247) is now recruiting and is estimated to be completed in 2026.^221^

### Poly-ADP-ribose polymerase 7 inhibitor RBN-2397

RBN-2397, a selective poly-ADP-ribose polymerase 7 (PARP7) inhibitor, acts as an indirect STING activator through reversing the negative regulation of PARP7 on type I IFN response, thereby restoring T cell-mediated anti-tumor immunity as well as suppressing tumor immune escape.^222^^,^^223^ Preclinical mouse tumor models have validated that oral dosing of RBN-2397 resulted in type I IFN-dependent induction of tumor-specific adaptive immune memory and complete tumor regression by inhibiting tumor cell proliferation and inducing durable immunity (Table 2).^77^^,^^224^ In addition to this, another PARP7 inhibitor, JAB-26766, had data from preclinical studies presented at the AACR Annual Meeting in 2024, clarifying that its combination with a STING agonist can synergistically activate the STING pathway.^201^ Pedretti et al. investigated the anti-tumor effects of the combination of the STING agonist diABZI and the PARP inhibitor olaparib in homologous recombination-deficient breast cancer. They found that the combination significantly enhanced immune cell infiltration, particularly NK cells, and demonstrated superior anti-tumor efficacy.^225^ Phase I study of RBN-2397 as monotherapy in patients with solid tumors has shown good tolerability and clinical activity with 1 patient with HR^+^, HER2^–^ breast cancer achieving a confirmed PR at 100 mg and 8 patients observed SD for more than 18 weeks (NCT04053673).^226^ Another phase Ib/II trial of RBN-2397 in combination with pembrolizumab in patients with squamous cell carcinoma of the lung (SCCL) was launched in 2022 to explore the ability of RBN-2397 to restore the response to ICIs in SCCL patients who had been previously treated with a PD-1/L1 inhibitor and have had disease progression after observed response (NCT05127590) (Figure 8).^227^

### SNX281

SNX281 is a novel small-molecule STING agonist with favorable pharmacokinetic properties, allowing for systemic administration via intravenous injection. In preclinical studies, SNX281 exhibited significant anti-tumor activity in mouse models, effectively eradicating certain tumors and inducing immunological memory (Table 2).^228^ Currently, SNX281 is undergoing a phase I clinical trial (NCT04609579) in patients with advanced solid tumors and lymphomas to evaluate its safety and efficacy both as a monotherapy and in combination with pembrolizumab. The final results have not yet been published (Figure 8).^229^

### STING inhibitors

While STING agonists have advanced into clinical trials for cancer immunotherapy, the role of STING inhibitors is of interest mainly in the treatment of autoinflammatory and autoimmune diseases. These inhibitors mainly act through three mechanisms: blocking palmitoylation, competing with endogenous ligands, or promoting STING degradation. Palmitoylation at Cys88 and Cys91 is critical for STING activation.^190^ Covalent inhibitors such as C-176, C-178, and H-151 irreversibly modify these residues to prevent activation.^230^^,^^231^^,^^232^^,^^233^ Endogenous nitro fatty acids (e.g., 10-NO_2_-OA) and itaconate derivatives such as 4-OI exhibit similar effects by alkylating Cys-91.^234^ 10-Nitro oleic acid (CXA-10) is one of the nitro fatty acids that reacts with the cysteine residues of STING to prevent palmitoylation of STING.^235^ Clinical trials of CXA-10 entered phase II, of which three were discontinued and one was completed. Other compounds, including Astin C, Compound 18, and SN-011, target the C-terminal ligand-binding pocket to competitively inhibit cGAMP-induced signaling.^16^^,^^236^^,^^237^ More recent inhibitors such as BB-Cl-amidine and LB244 covalently bind alternative cysteines (Cys-148 or Cys-292), blocking oligomerization.^238^^,^^239^ Additionally, natural products such as gelsevirine promote STING degradation via K48-linked ubiquitination.

It is worth noting that palbociclib, a CDK4/6 inhibitor, has recently been identified in research as also functioning as an STING inhibitor. Its mechanism of action is distinctive, as it directly targets the Y167 site of STING to prevent dimerization, thereby inhibiting its activation.^240^ Additionally, palbociclib can obstruct the formation of the STING-TBK1 complex through the G166 site, further impeding its activation.^241^ In models of DSS-induced colitis and Trex1^−/−^-mediated autoinflammatory diseases, palbociclib has been shown to reduce STING-mediated inflammation and tissue damage.^242^ This suggests that palbociclib holds potential therapeutic applications in STING inhibition, providing new insights and possible drug options for the treatment of autoinflammatory diseases.

Although most remain in preclinical development, these inhibitors offer compelling therapeutic potential and warrant further clinical investigation.

## Future perspectives

As an emerging target in cancer immunotherapy, STING plays a crucial role in every phase of the anti-tumor immune cycle. Recently, Xu and co-workers demonstrated that the cGAS-STING pathway activates transcription factor EB independently of the protein kinase TBK1, increasing lysosome biogenesis and improving pathogen clearance. This finding highlights that STING activation not only boosts immune responses but also influences the process of autophagy, which could have significant implications for cancer therapy.^243^^,^^244^ Although STING agonists have shown significant efficacy in preclinical models across various tumors, their clinical efficacy as monotherapy remains limited, with unclear mechanisms, posing substantial challenges to clinical application. This comprehensive review has provided deeper insights into the challenges facing the clinical application of STING agonists and potential solutions.

First, several strategies have been proposed to address intrinsic issues associated with STING agonists, such as inadequate cellular uptake, poor metabolic stability, limited targeting capability, and challenges with intratumoral delivery. Current advancements include enhancing the metabolic stability of CDNs, developing non-CDN STING agonists and ENPP1 inhibitors, incorporating adjuvants, and employing targeted delivery platforms such as ADCs and nanoparticles to increase targeting specificity and delivery efficiency.^245^^,^^246^ Looking forward, optimizing dosing regimens and administration routes for existing STING agonists, developing novel and stable STING agonists, and refining delivery strategies will be prioritized in clinical trials. Meanwhile, new avenues, such as developing STING phase separation inhibitors and VRAC channel agonists, are also being explored. Notably, further exploration of the relationship between STING conformational changes and activation, as well as the mechanistic role of pmSTING, holds promise for new STING agonist optimization strategies.

Second, the primary resistance of tumor cells significantly contributes to the ineffectiveness of STING agonists. Efforts are underway to develop broad-spectrum STING agonists to accommodate various hSTING mutations. Additionally, differences in STING expression levels across cancer types, cellular composition of the TME, as well as tumor cell STING suppression, alterations in expression levels, and immune evasion resulting from chronic activation all influence therapeutic outcomes. These factors highlight the importance of careful evaluation of treatment appropriateness when using STING agonists. Researchers are also investigating drugs such as DNMTis and MPS1 inhibitors to restore STING expression and exploring strategies for patient stratification based on tumor biology, staging, gene expression, CIN score, and the activation status of downstream STING molecules.

Finally, acquired resistance presents another significant obstacle, involving multiple factors such as upregulation of secondary immune checkpoint molecules, activation of immunosuppressive cells, inhibition of immune effector cells, and increased immunosuppressive factors. To address these mechanisms, STING agonists are being combined with other therapies, including surgery, chemotherapy, radiotherapy, PDT, CAR-T, ICIs, and antibodies targeting immunosuppressive factors,^179^ aiming to overcome resistance.

In summary, the evolution of next-generation STING-targeting strategies necessitates a sophisticated and multifaceted approach. This includes leveraging advanced screening methodologies, precision selection based on genomic variants, stratification based on tumor and microenvironment biomarkers, and reversing inhibition mechanisms. As we forge ahead, refining the timing and spacing of STING agonist administration, overcoming resistance through synergistic combination therapies, and designing biomarker-driven clinical trials will be of paramount importance. These coordinated efforts will not only deepen our comprehension of STING agonists but also chart a course toward more personalized and effective cancer treatments. By harmoniously integrating these strategies with ongoing research endeavors and clinical trials, we can envision a future where STING-based therapies stand as a beacon of hope for cancer patients.

The continuous advancement of mechanistic studies in preclinical models, the ongoing progression of clinical trials, and the development of physical, chemical, and molecular strategies to activate STING are accelerating our understanding of STING agonists’ principles and applications. We are confident that these efforts will lead to more effective STING-based therapies, bringing new hope to cancer patients.

## Funding and acknowledgments

This work was supported by the 10.13039/501100001809National Natural Science Foundation of China (grant 82373323 to T.Z., 82405119 to J.S., U24A20724 and 82273364 to X.L.), the 10.13039/501100012166National Key Research and Development Program of China (grant 2023YFC3504600 to T.Z.), the Heilongjiang Provincial Natural Science Foundation of China (grant YQ2024H022 to J.S.), the Research fees of Heilongjiang Provincial Research Institutes (CZKYF2025-1-4012 to T.Z.), the Heilongjiang Province New Era Longjiang Outstanding Doctoral Dissertation Project (grant LJYXL2022-068 to J.S.), and the Hai Yan Key Research Fund Project of Harbin Medical University Cancer Hospital (grant JJZD2024-21 to J.S.). The funders had no role in study design, data collection and analysis, decision to publish, or preparation of the manuscript.

## Author contributions

T.Z., J.T., and J.S. conceived and organized the review. J.S., Y.Z., N.Z., and T.Z. wrote the original draft. N.Z. and Y.Z. edited the table and figures. T.Z., J.T., J.S., E.S., L.M., G.K., and X.L. revised the manuscript. All authors contributed to the manuscript and approved the final version.

## Declaration of interests

The authors declare no competing interests.
